# Prediction of long-term mortality in patients with ischemic stroke based on clinical characteristics on the first day of ICU admission: An easy-to-use nomogram

**DOI:** 10.3389/fneur.2023.1148185

**Published:** 2023-04-14

**Authors:** Guangyong Jin, Wei Hu, Longhuan Zeng, Buqing Ma, Menglu Zhou

**Affiliations:** ^1^Department of Critical Care Medicine, Affiliated Hangzhou First People’s Hospital, Zhejiang University School of Medicine, Hangzhou, China; ^2^Department of Neurology, Affiliated Hospital of Hangzhou Normal University, Hangzhou, China

**Keywords:** intensive care unit, ischemic stroke, MIMIC-IV, mortality, nomogram, prediction model

## Abstract

**Background:**

This study aimed to establish and validate an easy-to-use nomogram for predicting long-term mortality among ischemic stroke patients.

**Methods:**

All raw data were obtained from the Medical Information Mart for Intensive Care IV database. Clinical features associated with long-term mortality (1-year mortality) among ischemic stroke patients were identified using least absolute shrinkage and selection operator regression. Then, binary logistic regression was used to construct a nomogram, the discrimination of which was evaluated by the concordance index (C-index), integrated discrimination improvement (IDI), and net reclassification index (NRI). Finally, a calibration curve and decision curve analysis (DCA) were employed to study calibration and net clinical benefit, compared to the Glasgow Coma Scale (GCS) and the commonly used disease severity scoring system.

**Results:**

Patients who were identified with ischemic stroke were randomly assigned into developing (*n* = 1,443) and verification (*n* = 646) cohorts. The following factors were associated with 1-year mortality among ischemic stroke patients, including age on ICU admission, marital status, underlying dementia, underlying malignant cancer, underlying metastatic solid tumor, heart rate, respiratory rate, oxygen saturation, white blood cells, anion gap, mannitol injection, invasive mechanical ventilation, and GCS. The construction of the nomogram was based on the abovementioned features. The C-index of the nomogram in the developing and verification cohorts was 0.820 and 0.816, respectively. Compared with GCS and the commonly used disease severity scoring system, the IDI and NRI of the constructed nomogram had a statistically positive improvement in predicting long-term mortality in both developing and verification cohorts (all with *p* < 0.001). The actual mortality was consistent with the predicted mortality in the developing (*p* = 0.862) and verification (*p* = 0.568) cohorts. Our nomogram exhibited greater net clinical benefit than GCS and the commonly used disease severity scoring system.

**Conclusion:**

This proposed nomogram has good performance in predicting long-term mortality among ischemic stroke patients.

## 1. Introduction

Globally, stroke remains the second-leading cause of death and the third-leading cause of death and disability combined (1). Stroke includes two types, namely hemorrhagic stroke and ischemic stroke, which accounted for 62.4% of all incident strokes in 2019 (1). Ischemic stroke is a devastating central nervous system disorder that can cause permanent neurological damage (2). Although important progress has been made in the treatment of ischemic stroke (3), the incidence and prognosis of ischemic stroke are still not optimistic (1). The annual number of stroke and stroke deaths increased significantly for several decades, placing a significant burden on the globe (1). Short-term mortality is a commonly used outcome in ischemic stroke research as it is readily measurable and can be influenced by acute interventions. The early identification of a poor prognosis based on prognostic risk factors is helpful in the treatment and management of ischemic stroke. On the other hand, long-term mortality is also an important outcome to consider as it reflects the overall burden of the disease and the impact of stroke on a patient’s overall health and wellbeing. Predicting long-term mortality can help guide decisions about ongoing medical management, such as the use of secondary prevention measures, and can also inform discussions about end-of-life care. Several features have been identified that are strongly associated with death in patients with ischemic stroke, namely, age (4–6), underlying dementia (7), underlying cancer (5, 6), white blood cells (WBC) (6), anion gap (AG) (8), mechanical ventilation (MV) (9), and osmotic therapy (10).

The construction of the mortality prediction model, based on prognostic risk factors and expressed in the visual nomogram, is helpful in assessing prognosis in clinical practice. In many studies, a nomogram was developed to assess short-term outcomes in ischemic stroke (6, 11). Unfortunately, there are limited literature studies reporting a nomogram for predicting long-term mortality among ischemic stroke patients (5, 6). A nomogram based on 536 stroke patients was shown to have better performance in predicting 360-day mortality from stroke (C-index = 0.804) (6); it should be noted that the nomogram was based on an OASIS scoring system, which means that the OASIS score must be completed before further use of the nomogram, and its utilization in clinical practice is complicated to some degree. Streamlined clinical prediction models predict disease prognosis based on few, easily accessible, low-cost predictors (12).

This study aimed to establish and validate an easy-to-use nomogram for predicting long-term mortality (1-year mortality) among ischemic stroke patients, based on conventional, accessible, and clinical characteristics on the 24 h after intensive care unit (ICU) admission from the Medical Information Mart for Intensive Care IV (MIMIC-IV) database.

## 2. Methods

### 2.1. Database

This study was conducted using the MIMIC-IV database (version 2.1), which included more than 70,000 admission records in the Beth Israel Deaconess Medical Center (BIDMC, Boston, MA) between 2008 and 2019. The version of the database was updated on 16 November 2022 and stored a wealth of clinical data, including demographics, vital signs, urine output, underlying diseases, disease severity assessment, laboratory information, medications, and interventions (13). All data in this database were de-identified, and it is impossible to identify specific patients. Therefore, the study is not considered a human subject research study and does not require the consent of the individual patient due to unidentified health information. Institutional review boards approved the establishment of the MIMIC-IV database at the Massachusetts Institute of Technology (MIT, Cambridge, MA) and BIDMC. The author, GJ, has completed the Human Subject Research Course (Certification Number: 46141344) and has therefore been granted access to the MIMIC-IV database.

### 2.2. Study population

**Figure 1:**
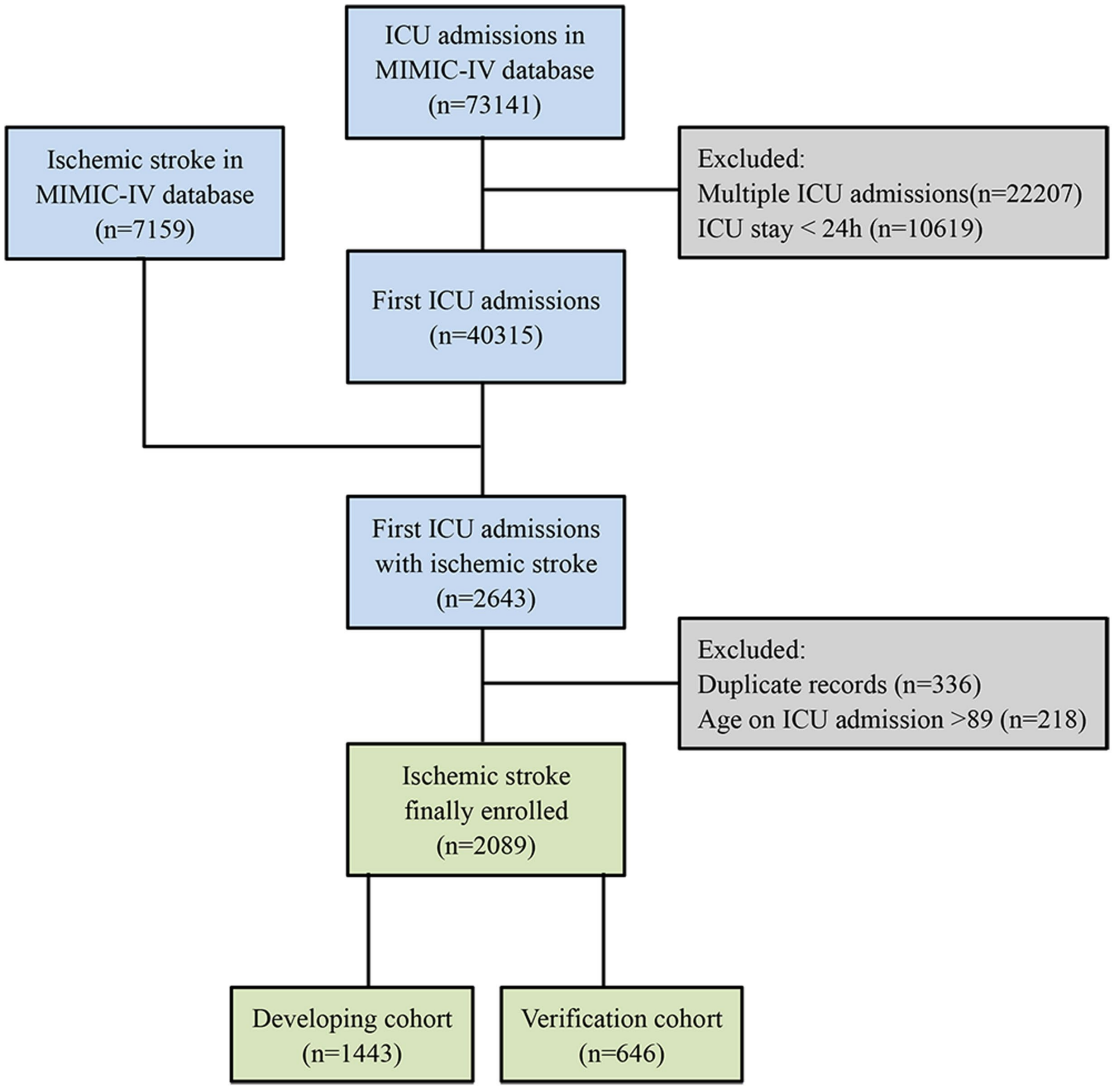
Flow chart of the patient selection process. MIMIC-IV, medical information mart for intensive care IV; ICU, intensive care unit.

Adults with ischemic stroke, who were admitted to the ICU for the first time and stayed in ICU for more than 1 day, were included in this study. The International Classification of Disease (ICD) (both version 9 and version 10) was used to identify ischemic stroke, as previously represented (14). The exclusion criteria were as follows: (a) second or more number of admission to ICU, (b) ICU stay was less than 24 h, and (c) if patients were over 89 years old, their age was set to 91 (i.e., all patients over 89 years were grouped into a group with an age value of 91, regardless of their chronological age), so we excluded patients over 89 years of age. Finally, 2089 ischemic stroke patients were identified and randomized to the developing cohort or verification cohort at a ratio of 7:3 (Figure 1).

### 2.3. Data extraction

All data were extracted using PostgreSQL tools (version 14.2.1). Demographic characteristics on ICU admission were identified as follows: age, gender, weight, first care unit, marital status, and race. Based on the time of hospital/ICU admission/discharge and death time, we calculated hospital length of stay (LOS) and ICU LOS and identified survival status for 1 year after ICU admission (1-year mortality), which was defined as both long-term mortality and a primary outcome. The following features were recorded on the first day of ICU stay by MIMIC-IV concepts: underlying diseases, Charlson comorbidity index (CCI), temperature, heart rate, respiratory rate, systolic blood pressure (SBP), oxygen saturation, glucose, urine output, WBC, hemoglobin, hematocrit, platelets, biochemical indicators (blood urea nitrogen (BUN), creatinine, sodium, and potassium), prothrombin time, partial thromboplastin time, international normalized ratio, medications (alteplase, antiplatelet, anticoagulation, furosemide, mannitol, and vasoactive agent), interventions (endovascular obstruction removal, invasive mechanical ventilation, supplemental oxygen, renal replacement therapy, and intracranial pressure monitor), Simplified Acute Physiology Score II (SAPS II), Acute Physiology Score III (APS III), Oxford Acute Severity of Illness Score (OASIS), Logistic Organ Dysfunction System (LODS), Sequential Organ Failure Assessment (SOFA), and Glasgow Coma Scale (GCS). Antiplatelet was defined as the use of aspirin, clopidogrel, or dipyridamole. Anticoagulation was defined as the use of heparin, warfarin, rivaroxaban, argatroban, apixaban, bivalirudin, or dabigatran. A vasoactive agent was defined as the use of norepinephrine, epinephrine, phenylephrine, dopamine, dobutamine, vasopressin, or milrinone. Data, measured several times during the first day of ICU hospitalization， were extracted minimum, maximum, and average values (if any) from the MIMIC concepts.

### 2.4. Construction of the Nomogram

R software (version 4.2.1) was applied to develop and verify the nomogram. The latent variables were filtered out from the developing cohort using least absolute shrinkage and selection operator (LASSO) regression with the “glmnet” R package, as previously described (15). Thereafter, taking survival status for 1 year after ICU admission as the dependent variable, logistic regression was utilized to identify the prognostic characteristics of 1-year mortality among ischemic stroke patients. The variance inflation factor was calculated using the “car” R package to detect the collinearity of the variables. The nomogram was constructed using the “rms” R data package to predict 1-year mortality of ischemic stroke patients after ICU admission. To assess the potential collinearity between variables included in our nomogram, we utilized the variance inflation factor (VIF).

### 2.5. Verification of the Nomogram

We compared the performance of the nomogram with other logistic regression models based on the disease severity scoring system. The “pROC” R package was used to obtain the concordance index (C-index) in both developing and verification cohorts. The improvement of prediction performance on different models was evaluated using the “PredictABEL” R package, by integrated discrimination improvement (IDI) and the index of net reclassification (NRI) (12). Then, we applied the “val.prob” function in the “rms” R data package to generate calibration curves for the evaluation of model accuracy on both groups. Decision curve analysis (DCA) was performed with the “rmda” R data package for the developing cohort to assess the clinical benefits of a developed nomogram, compared to several logistic regression models based on the disease severity scoring system.

### 2.6. Statistical analysis

A histogram was applied to detect outliers, which were winsorized (replace cuts 0.5 and 99.5%) in Stata software (version 17.0). Multiple imputations were performed to fill in missing values. All patients were randomized to either the developing cohort or the verification cohort, and the clinical characteristics of both cohorts were statistically analyzed to assess the scientificity and rationality of the grouping. The normality of continuous variables was tested using the skewness and kurtosis test. Then, nonnormally distributed continuous variables were compared to find differences between the two cohorts using the Mann–Whitney *U*-test, and the results were expressed as median and the interquartile range (IQR). Categorical variables were expressed as numbers or percentages and were tested using the chi-square test. A *p*-value of <0.05 was considered to be statistically significant.

## 3. Results

### 3.1. Baseline characteristics

Demographic and clinical characteristics of all patients with ischemic stroke in the developing cohort (*n* = 1,443) and verification cohort (*n* = 646) are described in Table 1. No statistically significant differences in baseline features of ischemic stroke patients in the developing and verification cohorts were seen, including demographics, underlying diseases, disease severity, vital signs, laboratory indicators, medications, and interventions (*p* > 0.05), indicating that the entire research objects were distributed scientifically and reasonably.

**Table 1:** Clinical characteristics of the patients with ischemic stroke.

Characteristics	All patients (*N* = 2089)	Developing cohort (*n* = 1,443)	Verification cohort (*n* = 646)	*p* value
Age, median (IQR)	68.92 (57.33, 78.86)	69.05 (57.88, 78.94)	68.42 (56.50, 78.57)	0.515
Female, no. (%)	993 (47.53)	682 (47.26)	311 (48.14)	0.710
Weight, median (IQR) (kg)	78.10 (66.50, 93.50)	78.00 (66.90, 93.20)	79.20 (65.90, 93.90)	0.982
*Race, no. (%)*				
White	1242 (59.45)	854 (59.18)	388 (60.06)	0.119
Hispanic	74 (3.54)	49 (3.40)	25 (3.87)	
Black	223 (10.67)	160 (11.09)	63 (9.75)	
ASIAN	60 (2.87)	33 (2.29)	27 (4.18)	
Other	490 (23.46)	347 (24.05)	143 (22.14)	
*Marital status, no. (%)*				
Married	894 (42.80)	604 (41.86)	290 (44.89)	0.299
Single	516 (24.70)	356 (24.67)	160 (24.77)	
Widowed	217 (10.39)	163 (11.30)	54 (8.36)	
Divorced	146 (6.99)	99 (6.86)	47 (7.28)	
Other	316 (15.13)	221 (15.32)	95 (14.71)	
*First care unit, no. (%)*				
Medical ICU	190 (9.10)	132 (9.15)	58 (8.98)	0.886
Surgical ICU	663 (31.74)	456 (31.60)	207 (32.04)	
Medical ICU/Surgical ICU	101 (4.83)	75 (5.20)	26 (4.02)	
Neuro Surgical ICU	284 (13.60)	192 (13.31)	92 (14.24)	
Trauma Surgical ICU	223 (10.67)	152 (10.53)	71 (10.99)	
Other ICU	628 (30.06)	436 (30.21)	192 (29.72)	
*Underlying diseases, no. (%)*				
Myocardial Infarct	327 (15.65)	222 (15.38)	105 (16.25)	0.613
Congestive Heart Failure	467 (22.36)	327 (22.66)	140 (21.67)	0.616
Peripheral Vascular Disease	275 (13.16)	193 (13.37)	82 (12.69)	0.670
Dementia	69 (3.30)	53 (3.67)	16 (2.48)	0.157
Chronic Pulmonary Disease	377 (18.05)	249 (17.26)	128 (19.81)	0.160
Rheumatic Disease	57(2.73)	40(2.77)	17 (2.63)	0.856
Peptic Ulcer Disease	33 (1.58)	22 (1.52)	11 (1.70)	0.763
Mild Liver Disease	111 (5.31)	72 (4.99)	39 (6.04)	0.324
Severe Liver Disease	35 (1.68)	26 (1.80)	9 (1.39)	0.501
Renal Disease	321 (15.37)	220 (15.25)	101 (15.63)	0.820
Diabetes without chronic complication	573 (27.43)	389 (26.96)	184 (28.48)	0.470
Diabetes with chronic complication	175 (8.38)	131 (9.08)	44 (6.81)	0.084
Paraplegia	991 (47.44)	696 (48.23)	295 (45.67)	0.277
Malignant Cancer	181 (8.66)	125 (8.66)	56 (8.67)	0.996
Metastatic Solid Tumor	74 (3.54)	52 (3.60)	22 (3.41)	0.821
Acquired Immune Deficiency Syndrome	5 (0.24)	2 (0.14)	3 (0.46)	0.159
**Charlson Comorbidity Index, median (IQR)**	7.00 (5.00, 8.00)	7.00 (5.00, 8.00)	7.00 (5.00, 9.00)	0.453
*Disease severity scoring system, median (IQR)*				
Firstday GCS^a^	12.00 (8.00, 14.00)	12.00 (8.00, 14.00)	12.00 (8.00, 14.00)	0.898
Firstday SOFA	4.00 (2.00, 6.00)	4.00 (2.00, 7.00)	4.00 (2.00, 6.00)	0.867
Firstday LODS	4.00 (2.00, 7.00)	4.00 (2.00, 7.00)	4.00 (2.00, 7.00)	0.831
Firstday OASIS	33.00 (26.00, 40.00)	33.00 (26.00, 40.00)	33.00 (26.00, 40.00)	0.573
Firstday APS III	42.00 (30.00, 61.00)	42.00 (30.00, 61.00)	43.00 (30.00, 62.00)	0.901
Firstday SAPS II	32.00 (25.00, 41.00)	32.00 (25.00, 41.00)	32.00 (25.00, 42.00)	0.571
*Vital Indicators, median (IQR)*				
Temperature (°C)^b^	37.28 (37.06, 37.83)	37.28 (37.06, 37.80)	37.33 (37.00, 37.90)	0.482
Heart Rate (beats/min)^b^	99.00 (86.00, 11,300)	99.00 (87.00, 11,400)	98.00 (85.00, 11,200)	0.213
Respiratory Rate (breaths/min)^c^	18.65 (16.84, 21.05)	18.64 (16.80, 21.10)	18.74 (17.00, 20.94)	0.698
SBP (mmHg)^b^	161.00 (145.00, 178.00)	160.00 (144.00, 178.00)	162.00 (147.00, 179.00)	0.171
Oxygen Saturation (%)^a^	93.00 (91.00, 95.00)	93.00 (91.00, 95.00)	93.00 (91.00, 95.00)	0.937
Glucose (mmol/L)^a^	5.83 (4.94, 7.06)	5.83 (4.89, 7.11)	5.78 (4.94, 7.00)	0.765
Firstday Urine Output (L)	1.60 (1.04, 2.35)	1.61 (1.04, 2.37)	1.58 (1.03, 2.32)	0.547
*Laboratory Indicators, median (IQR)*				
White Blood Cells (K/uL)^b^	11.90 (8.80, 15.90)	11.90 (8.70, 16.00)	11.85 (9.00, 15.60)	0.947
Hemoglobin (g/dL)^a^	11.60 (9.70, 13.10)	11.50 (9.60, 13.10)	11.80 (9.80, 13.20)	0.233
Hematocrit (%)^a^	34.90 (29.50, 39.10)	34.70 (29.40, 39.00)	35.20 (29.70, 39.40)	0.332
Platelets (K/uL)^a^	195.00 (149.00, 252.00)	196.00 (149.00, 254.00)	194.00 (149.00, 245.00)	0.362
BUN (mg/dL)^b^	6.78 (5.00, 9.64)	6.43 (4.64, 9.64)	6.78 (5.00, 9.28)	0.487
Creatinine (μmmol/L)^b^	88.40 (70.72, 114.92)	88.40 (70.72, 114.92)	88.40 (70.72, 114.92)	0.082
Sodium (mEq/L)^b^	140.00 (138.00, 143.00)	140.70 (138.00, 143.00)	140.00 (138.00, 143.00)	0.524
Potassium (mEq/L)^b^	4.30 (3.90, 4.70)	4.30 (3.90, 4.70)	4.20 (3.90, 4.70)	0.346
Calcium (mEq/L)^a^	2.13 (1.98, 2.23)	2.13 (1.98, 2.23)	2.13 (1.98, 2.25)	0.669
Chloride (mEq/L)^b^	106.00 (103.00, 109.00)	106.00 (102.00, 109.00)	106.00 (103.00, 109.00)	0.395
Prothrombin Time (sec)^b^	13.10 (11.90, 15.30)	13.20 (11.90, 15.50)	13.00 (11.90, 14.90)	0.134
PTT (sec)^b^	30.40 (26.80, 40.60)	30.50 (26.80, 40.70)	30.35 (26.90, 40.60)	0.871
INR^b^	1.20 (1.10, 1.40)	1.20 (1.10, 1.40)	1.20 (1.10, 1.38)	0.126
Anion Gap (mmol/L)^a^	13.00 (12.00, 15.00)	13.00 (12.00, 15.00)	13.00 (11.00, 15.00)	0.136
Bicarbonate (mmol/L)^a^	22.00 (20.00, 24.00)	22.00 (20.00, 24.00)	22.00 (19.42, 24.00)	0.127
*Medications and interventions, no. (%)*				
Endovascular obstruction Removal	192 (9.19)	131 (9.08)	61 (9.44)	0.790
Alteplase	38 (1.82)	24 (1.66)	14 (2.17)	0.426
Antiplatelet	559 (26.76)	394 (27.30)	165 (25.54)	0.400
Anticoagulation	688 (32.93)	477 (33.06)	211 (32.66)	0.860
Furosemide	93 (4.45)	68 (4.71)	25 (3.87)	0.388
Mannitol	83 (3.97)	55 (3.81)	28 (4.33)	0.572
Vasoactive agent	515 (24.65)	355 (24.53)	161 (24.92)	0.848
Invasive mechanical ventilation	756 (36.19)	524 (36.31)	232 (35.91)	0.860
Supplemental oxygen	794 (38.01)	552 (38.25)	242 (37.46)	0.730
Renal replacement therapy	55 (2.63)	39 (2.70)	16 (2.48)	0.766
Intracranial pressure monitor	85 (4.07)	57 (3.95)	28 (4.33)	0.681
*Outcomes*				
28-Day Mortality (%)	457 (21.88)	311 (21.55)	146 (22.60)	0.592
ICU Mortality (%)	269 (12.88)	172 (11.92)	97 (15.02)	0.051
Hospital mortality (%)	378 (18.09)	246 (17.05)	132 (20.43)	0.063
ICU LOS (days)	3.81 (1.98, 7.78)	3.85 (2.00, 7.89)	3.54 (1.96, 7.52)	0.589
Hospital LOS (days)	9.04 (5.03, 17.54)	9.18 (5.03, 17.27)	8.95 (5.03, 17.70)	0.558

ICU, Intensive Care Unit; IQR, interquartile range; GCS, Glasgow Coma Scale; APS III, Acute Physiology Score III; SOFA, Sequential Organ Failure Assessment; LODS, Logistic Organ Dysfunction System; SAPS II, Simplified Acute Physiology Score II; OASIS, Oxford Acute Severity of Illness Score; SBP, Systolic Blood Pressure; BUN, Blood Urea Nitrogen; PTT, Partial Thromboplastin Time; INR, International Normalized Ratio; LOS, Length of Stay.

Antiplatelet was defined as the use of aspirin, clopidogrel, or dipyridamole within 24 h after ICU admission. Anticoagulation was defined as the use of heparin, warfarin, rivaroxaban, argatroban, apixaban, bivalirudin, or dabigatran within 24 h after ICU admission. A vasoactive agent was defined as the use of norepinephrine, epinephrine, phenylephrine, dopamine, dobutamine, vasopressin, or milrinone within 24 h after ICU admission. The disease severity scoring system, vital indicators, laboratory indicators, and interventions were evaluated within 24 h after ICU admission.

a The min value of indicators on the first day of ICU stay.

b The max value of indicators on the first day of ICU stay.

c The mean value of indicators on the first day of ICU stay.

### 3.2. Features identification and construction of the Nomogram

**Figure 2:**
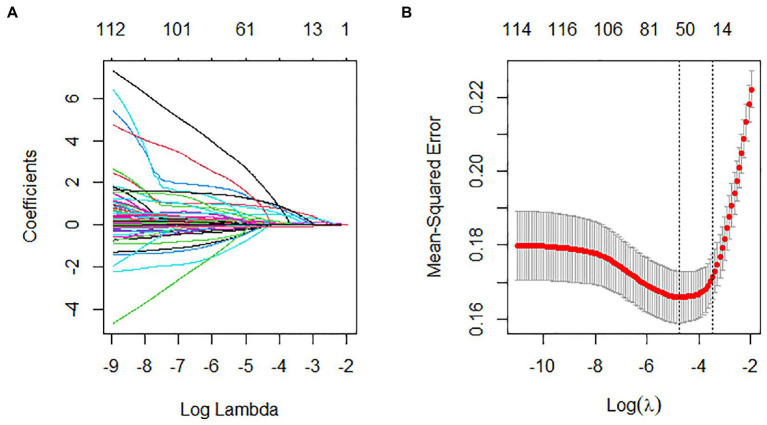
Selection of clinical features by least absolute shrinkage and selection operator (LASSO) regression and 10-fold cross-validation. **(A)** A visual plot of the relationship between coefficients for features and the lambda. The coefficient of each feature gradually tended to zero as the lambda value increased and **(B)** curve of 10-fold cross-validation in the LASSO regression. The dotted vertical line on the left reflects the number of features and optimal log (lambda) corresponding to the smallest mean squared error. With one standard error criterion of optimal log (lambda), the dotted vertical line on the right indicates that the model constructed with 13 variables was relatively accurate and simple (λ = 0.03114428). λ, lambda.

**Figure 3:**
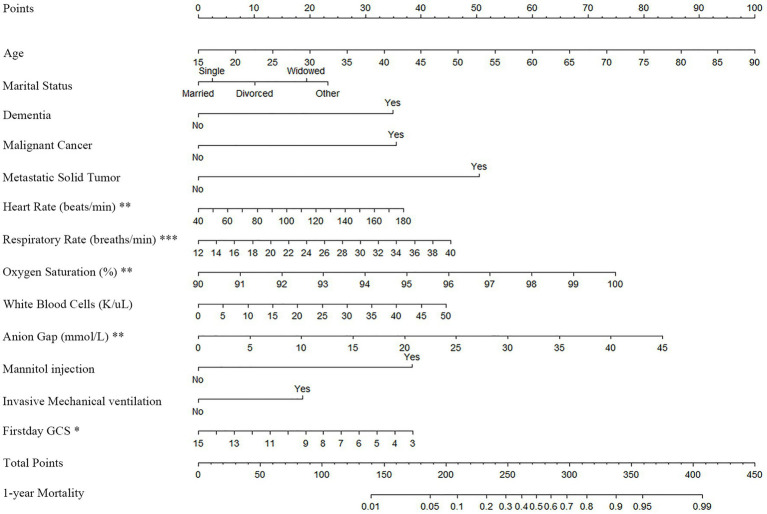
Nomogram for predicting 1-year mortality among ischemic stroke patients. A specific patient can be scored according to the items in the nomogram, and the 1-year mortality is assessed according to the total points obtained.

LASSO regression and 10-fold cross-validation were utilized to identify clinical factors associated with 1-year mortality in adults with ischemic stroke (Figures 2A,B). A total of 18 underlying clinical features, namely, age on ICU admission, marital status (married, single, widowed, divorced, or other), dementia (yes), malignant cancer (yes), metastatic solid tumor (yes), CCI, heart rate (maximum value), respiratory rate (average value), oxygen saturation (maximum value), hemoglobin (minimum value), hemoglobin (maximum value), WBC (maximum value), anion gap (maximum value), BUN (minimum value), BUN (maximum value), mannitol injection (yes), invasive mechanical ventilation (yes), and GCS (minimum value), were selected for further binary multivariate regression analysis. In total, 13 of them were conclusively demonstrated as independent predictors of long-term mortality among ischemic stroke patients (*p* < 0.05) (Table 2). The nomogram was developed using selected clinical features (Figure 3).

**Table 2:** Multivariable logistic regression analyses of independent risk factors of the 1-year mortality in patients with ischemic stroke in the developing cohort.

Variables	OR	95% CI	*p*-value	VIF
Age	1.047	1.036–1.058	<0.001	1.287
Marital Status (Single)	1.090	0.763–1.554	0.633	1.269
Marital Status (Widowed)	1.948	1.280–2.967	0.002	1.257
Marital Status (Divorced)	1.413	0.818–2.406	0.208	1.113
Marital Status (Other)	2.221	1.530–3.228	<0.001	1.248
Dementia (Yes)	3.313	1.756–6.387	<0.001	1.052
Malignant Cancer (Yes)	3.388	2.021–5.722	<0.001	1.312
Metastatic Solid Tumor (Yes)	5.643	2.429–13.812	<0.001	1.342
Heart Rate (beats/min)^b^	1.009	1.002–1.016	0.007	1.123
Respiratory Rate (breaths/min)^c^	1.057	1.015–1.101	0.007	1.159
Oxygen Saturation (%)^b^	1.292	1.107–1.525	0.002	1.134
White Blood Cells (K/uL)	1.031	1.009–1.054	0.006	1.189
Anion Gap (mmol/L)^b^	1.066	1.031–1.102	<0.001	1.112
Mannitol injection (Yes)	3.723	2.001–7.090	<0.001	1.022
Invasive Mechanical ventilation (Yes)	1.902	1.416–2.558	<0.001	1.278
Firstday GCS^a^	0.896	0.864–0.929	<0.001	1.129

OR, odd ratio; CI, confidence interval; GCS, Glasgow Coma Scale; VIF, variance inflation factor.

a The minimum value of indicators on the first day of ICU stay.

b The maximum value of indicators on the first day of ICU stay.

c The mean value of indicators on the first day of ICU stay.

### 3.3. Evaluation and verification of the Nomogram

**Figure 4:**
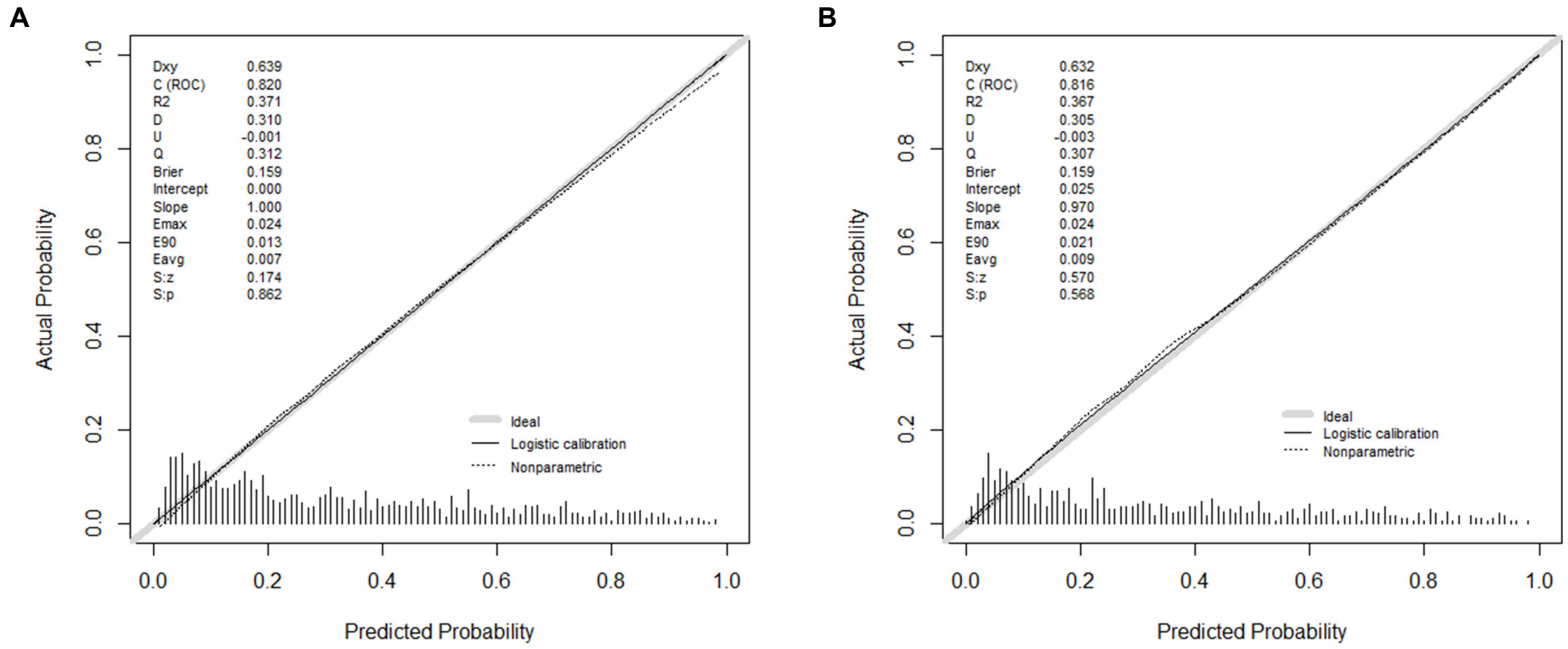
Calibration curve of constructed nomogram in the developing set **(A)** and verification set **(B)**. The actual mortality was consistent with the predicted mortality in the developing (*p* = 0.862) and verification (*p* = 0.568) cohorts.

The C-index of the nomogram in the developing and verification cohorts was 0.820 (95% Confidence Interval, 95% CI: 0.797, 0.842) and 0.816 (95% CI: 0.782, 0.851), respectively. This suggests that the established nomogram has good accuracy in predicting the long-term prognosis of ischemic stroke. In both datasets, the C-index of the constructed nomogram was higher than that of the commonly used clinical scoring system (Table 3). Compared with GCS and the commonly used disease severity scoring system, the IDI and NRI of the constructed nomogram had a statistically positive improvement in predicting long-term mortality in both developing and verification cohorts (all with *p* < 0.001) (Table 4). As a result of the calibration curve, the actual 1-year mortality of patients with ischemic stroke was consistent with the 1-year mortality predicted by the developed nomogram, and there was no statistically significant difference in the developing (*p* = 0.862) and verification (*p* = 0.568) cohorts (Figures 4A,B).

**Table 3:** C-index of nomogram and critical care scoring systems for predicting 1-year mortality in ischemic stroke patients.

Models	Developing cohort	Verification cohort		
	C-index	95% CI	C-index	95% CI
Nomogram	0.820	0.797–0.842	0.816	0.782–0.851
GCS	0.654	0.622–0.685	0.693	0.647–0.739
SOFA	0.708	0.680–0.735	0.688	0.645–0.730
APS III	0.742	0.715–0.769	0.741	0.701–0.781
LODS	0.731	0.704–0.758	0.736	0.696–0.776
SAPS II	0.760	0.735–0.786	0.754	0.715–0.792
OASIS	0.733	0.706–0.759	0.748	0.708–0.787

C-index, concordance index; CI, confidence interval; GCS, Glasgow coma score; SOFA, sequential organ failure assessment; APS III, acute physiology score III; LODS, logistic organ dysfunction system; SAPS II, simplified acute physiology score II; OASIS, oxford acute severity of illness score.

**Table 4:** Comparison of NRI and IDI models for predicting the 1-year mortality in patients with ischemic stroke.

Index	Developing cohort			Verification cohort		
	Estimate	95% CI	*p* value	Estimate	95% CI	*p* value
NRI (vs. GCS)	0.529	0.454–0.604	<0.001	0.265	0.160–0.369	<0.001
NRI (vs. SOFA)	0.473	0.395–0.551	<0.001	0.650	0.536–0.764	<0.001
NRI (vs. APS III)	0.361	0.288–0.434	<0.001	0.281	0.175–0.387	<0.001
NRI (vs. LODS)	0.278	0.202–0.353	<0.001	0.251	0.146–0.356	<0.001
NRI (vs. SAPS II)	0.247	0.174–0.319	<0.001	0.249	0.148–0.349	<0.001
NRI (vs. OASIS)	0.305	0.234–0.375	<0.001	0.195	0.100–0.290	<0.001
IDI (vs. GCS)	0.207	0.184–0.231	<0.001	0.157	0.120–0.193	<0.001
IDI (vs. SOFA)	0.200	0.174–0.226	<0.001	0.206	0.170–0.241	<0.001
IDI (vs. APS III)	0.151	0.126–0.175	<0.001	0.137	0.100–0.173	<0.001
IDI (vs. LODS)	0.156	0.131–0.182	<0.001	0.142	0.106–0.178	<0.001
IDI (vs. SAPS II)	0.120	0.097–0.143	<0.001	0.122	0.088–0.155	<0.001
IDI (vs. OASIS)	0.144	0.121–0.166	<0.001	0.126	0.094–0.158	<0.001

NRI, net reclassification index; IDI, integrated discrimination improvement; CI, confidence interval; GCS, Glasgow coma score; SOFA, sequential organ failure assessment; APS III, acute physiology score III; LODS, logistic organ dysfunction system; SAPS II, simplified acute physiology score II; OASIS, oxford acute severity of illness score. Cutoff: 0, 0.2, 0.4, 1.

### 3.4. Clinical value of the Nomogram

**Figure 5:**
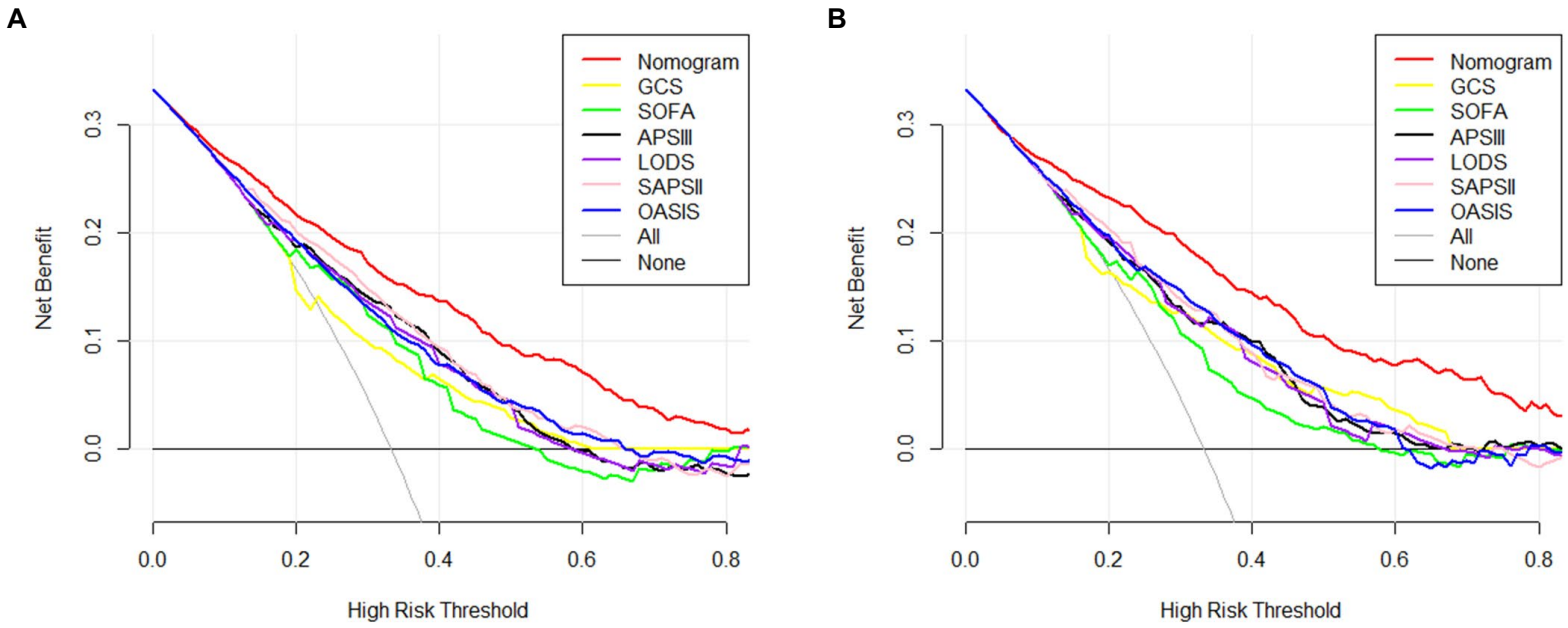
The decision curves analysis of the constructed nomogram and models based on common clinical scoring systems in the training set **(A)** and validation set **(B)**. In both sets, the red line represents the results of constructed nomogram and the best performance was achieved by our nomogram. GCS, Glasgow coma score; SOFA, sequential organ failure assessment; APS III, acute physiology score III; LODS, logistic organ dysfunction system; SAPS II, simplified acute physiology score II; OASIS, oxford acute severity of illness score.

DCA curve analysis was utilized to assess the clinical benefit and value of the constructed nomogram in the developing cohort. As shown in Figure 5, the red line represents the developed nomogram, which exhibited greater net clinical benefit than GCS and the commonly used disease severity scoring system.

## 4. Discussion

In this study, 13 independent risk factors for 1-year mortality among ischemic stroke patients were identified using LASSO regression, 10-fold cross-validation method, and binary multivariate logistic regression, namely, age on ICU admission, marital status (married, single, widowed, divorced, or other), dementia (yes), malignant cancer (yes), metastatic solid tumor (yes), heart rate (maximum value), respiratory rate (average value), oxygen saturation (maximum value), WBC (maximum value), anion gap (maximum value), mannitol injection (yes), invasive mechanical ventilation (yes), and GCS (minimum value), which were utilized to construct a nomogram for predicting long-term mortality (1-year mortality) among ischemic stroke patients. The results of the C-index demonstrated that the established nomogram had good discrimination and could better distinguish the survival status of ischemic stroke patients 1 year after ICU admission. In addition, the results of NRI and IDI showed that our nomogram, as a new mortality prediction model, had a significant improvement in predicting long-term mortality among ischemic stroke patients compared with GCS and the commonly used traditional disease severity scoring system. The calibration curves in both cohorts suggest that our nomogram was well calibrated, suggesting that as a predictive model, our nomogram was robust and stable. DCA revealed that our nomogram was superior to those based on GCS and the commonly used traditional disease severity scoring system with regard to net clinical benefits.

A nomogram is a visualization method for clinical predictive models that are widely used in the field of ischemic stroke. For example, cognitive impairment is one of the common symptoms after ischemic stroke and can be accurately predicted by the constructed nomogram (16, 17). Radiomics can be used to identify quantitative imaging indexes, which have the advantage of non-invasiveness; a novel nomogram, including clinical features and radiomics features, was constructed to predict neurological outcomes in ischemic stroke and exhibited good performance (18). Zheng et al. (19) developed a simple nomogram for evaluating neurological outcomes of acute ischemic stroke treated with intravenous thrombolysis, and external validation demonstrated the robustness of the predictive model. Hyperglycemia was associated with poor stroke functional outcomes and was used to construct a nomogram, which demonstrated good clinical utility in predicting functional outcomes (20). Notably, a nomogram has also been utilized to predict mortality in patients with ischemic stroke (5, 6). Based on four clinical features and a nutritional risk score, a constructed nomogram was developed to reliably predict in-hospital mortality among acute stroke (21).

Many traditional disease severity scoring systems were used to elucidate disease prognosis or severity in clinical settings (22, 23). The GCS score, commonly used to assess coma, is part of the ischemic stroke score, and a low GCS score was helpful in predicting in-hospital mortality among acute ischemic stroke patients (24). This study found that the lowest GCS after 24 h of ICU admission was a risk factor, which was associated with long-term prognosis among ischemic stroke patients. In addition, the SOFA score was commonly utilized to evaluate organ function in patients with sepsis and was rarely used in ischemic stroke prognosis. Nevertheless, the SOFA score has been shown to accurately predict the prognosis of severe acute ischemic stroke (25). OASIS, including ten assessments, facilitated clinical application and was excellent at predicting mortality in the neurological ICU (23). Interestingly, despite the larger proportion of neurological score weight in LODS, our nomogram was superior in predicting long-term prognosis among ischemic stroke patients. The APS III score, similar to SAPS II, was second to our nomogram in its ability to predict long-term mortality among ischemic stroke patients in this study (C-index = 0.761). In terms of discrimination, calibration, and clinical net benefit, the nomogram established in this study has better performance in predicting 1-year mortality among ischemic stroke patients, compared to the scoring system mentioned above.

This study considered that some demographic factors and underlying diseases were associated with the 1-year mortality among ischemic stroke patients. Stroke was known to be associated with aging (6). This study found that an increase in age was a risk factor for adverse outcomes among ischemic stroke patients, as a previous study described (4). Conversely, better outcomes were more common in younger ischemic stroke patients (26). With an increase in age, chronic inflammation and ischemic injury in the brain of older adults were associated with worse outcomes among stroke, in which chronic immune activation and inflammation lead to further tissue loss and neurodegeneration (27); aging altered the immune microenvironment and neutrophil phenotype, exacerbating neutrophil pathogenicity in ischemia: neutrophils entering the inflammatory tissue could exhibit retrograde movement and re-enter the lumen of the vessel and remain further in downstream organs, increasing the severity of neurologic deficits and poor prognosis (28). Married ischemic stroke patients were found to have a better prognosis, while unmarried people had poorer post-stroke outcomes in this study, as a previous study described (29). Marriage provides a direct form of social support that reduces the risk of unhealthy behaviors (30) and therefore may provide more stable behavioral and psychosocial resources for the prevention and treatment of illness (31). Expectably, the effect of marital status on stroke prognosis will be described in a new and ongoing meta-analysis (PROSPERO ID: CRD42022295975). Dementia placed a heavy burden on the healthcare system. Stroke may lead to vascular dementia, while patients with pre-stroke dementia have poorer outcomes. A multicenter retrospective study demonstrated that people with pre-stroke dementia had a higher proportion of complications during hospitalization and a higher likelihood of dying during hospitalization; pre-existing cognitive impairment limits the ability of stroke to advocate for care needs (7). Underlying malignant cancers and metastatic solid tumors are also associated with increased mortality among stroke patients. This study demonstrated that cancer can predict a poor prognosis for ischemic stroke (5). Tumors often develop in areas of chronic inflammation, which promotes tumor growth and increases thrombosis risk (32).

Prognosis is deeply influenced by vital signs and laboratory metrics among ischemic stroke patients. Few studies have examined the relationship between heart rate and long-term prognosis among ischemic stroke patients. Our study suggested a positive correlation between heart rate and long-term mortality. Increased heart rate in stroke patients may indicate sympathetic overactivity and activation, which triggers systemic immunosuppression and is the main risk factor for death (33). A retrospective analysis of 831 patients with acute ischemic stroke found that the heart rate was correlated with mortality within 1 year after stroke onset, with maximum heart rate having the highest predictive power (34). Our nomogram also uses the maximum heart rate as one of the predictors. To the best of our knowledge, there are no studies specifically elaborating that respiratory rate predicts 1-year mortality among ischemic stroke patients. Patients who died in the hospital had a higher respiratory rate compared with those who survived acute ischemic stroke (35), which supports respiratory rate as a predictor of stroke mortality in our nomogram. The increased maximum oxygen saturation is associated with long-term mortality in our nomogram. Low oxygen saturation is generally thought to indicate inadequate oxygen delivery, while high oxygen saturation contributes to oxygen delivery. Indeed, the application of oxygen may cause hyperxemia and promote the formation of reactive oxygen species (36). Excessive oxygen delivery may expose patients to hyperoxic states, leading to potential iatrogenic harm, including lung injury, increased systemic vascular resistance, decreased heart rate due to increased parasympathetic tone, decreased cardiac output, decreased cerebral blood flow, and central nervous system toxicity (37). Many studies have demonstrated the ability of WBC to predict short-term mortality from ischemic stroke (38). Few studies discussed the role of WBC in predicting long-term mortality among ischemic stroke patients. Plasma AG has been demonstrated to predict in-hospital outcomes among acute ischemic stroke patients (AUC: 0.631) (8).

The need for MV, suggesting possible respiratory failure and indicating seriousness, was found to be associated with adverse outcomes (9). Neurological deterioration could be caused by cerebral edema (39). Osmotherapy is reasonable in patients with clinical deterioration of cerebral infarction-related cerebral edema. Mannitol injection was associated with the risk of mortality among cerebral edema due to acute ischemic stroke (10). The need for mannitol suggests the possibility of cerebral edema, which may be more severe. In addition, mannitol administration may cause osmotic substances to be transferred to the brain tissue through the disrupted blood–brain barrier, worsening edema (40). Mannitol is thought to promote apoptosis, inflammatory response, and oxidative stress.

There are some limitations in this study. First, MIMIC-IV is a large database with the potential for data bias and input errors. We have adopted methods to detect and handle outliers. Second, this is a retrospective study, and we look forward to further prospective studies validating the clinical utility of our nomogram. In addition, due to the limitations of the database, some important features were not included in this study, namely neuroimaging and electrophysiological examination. In the end, a nomogram was only verified internally. External validation is required to verify the performance of the nomogram.

## 5. Conclusion

The following factors were found to be significantly associated with long-term mortality (1-year mortality) among ischemic stroke patients, including age on ICU admission, marital status, underlying dementia, underlying malignant cancer, underlying metastatic solid tumor, heart rate, respiratory rate, oxygen saturation, WBC, anion gap, mannitol injection, invasive mechanical ventilation, and GCS. Logistic regression was applied to develop and verify a prognostic nomogram for predicting long-term mortality among ischemic stroke patients. The results of the C-index, NRI, IDI, and DCA revealed that the developed nomogram demonstrates satisfactory discrimination, calibration, and net clinical benefit. Further prospective studies and external validation are needed to validate the performance of the constructed nomogram.

## Data availability statement

The data analyzed in this study was obtained from the Medical Information Mart for Intensive Care IV (MIMIC-IV) database, the following licenses/restrictions apply: To access the files users must be credentialed users, complete the required training (CITI Data or Specimens Only Research) and sign the data use agreement for the project. Requests to access these datasets should be directed to PhysioNet, https://physionet.org/, doi: 10.13026/rrgf-xw32.

## Ethics statement

Ethical review and approval was not required for the study on human participants in accordance with the local legislation and institutional requirements. Written informed consent for participation was not required for this study in accordance with the national legislation and the institutional requirements.

## Author contributions

GJ and MZ: study design. GJ and LZ: data extraction. BM and MZ: data curation. GJ and WH: statistical analysis. GJ, WH, LZ, BM, and MZ: writing—original draft preparation. WH and BM: writing—review and editing. WH: supervision and project administration. WH, LZ, and MZ: resources and funding acquisition. All authors contributed to the article and approved the submitted version.

## Funding

This study was supported by grants from the Zhejiang Medical and Health Science and Technology Project (grant: 2021KY894), the Project of Hangzhou Science and Technology (grant: 20201203B198), and the Construction Fund of Medical Key Disciplines of Hangzhou (grant: OO20200485).

## Conflict of interest

The authors declare that the research was conducted in the absence of any commercial or financial relationships that could be construed as a potential conflict of interest.

## Publisher’s note

All claims expressed in this article are solely those of the authors and do not necessarily represent those of their affiliated organizations, or those of the publisher, the editors and the reviewers. Any product that may be evaluated in this article, or claim that may be made by its manufacturer, is not guaranteed or endorsed by the publisher.

## Figures and Tables

**Table:** 

ICU	Intensive Care Unit
IQR	interquartile range
OR	odd ratio
CI	confidence interval
C-index	concordance index
NRI	net reclassification index
IDI	integrated discrimination improvement
DCA	decision curves analysis
LASSO	least absolute shrinkage, and selection operator
MIMIC-IV	medical information mart for intensive care IV
ICD	international classification of disease
CCI	Charlson comorbidity index
WBC	white blood cells
AG	anion gap
MV	mechanical ventilation
GCS	Glasgow Coma Scale
APS III	Acute Physiology Score III
SOFA	Sequential Organ Failure Assessment
LODS	Logistic Organ Dysfunction System
SAPS II	Simplified Acute Physiology Score II
OASIS	Oxford Acute Severity of Illness Score
SBP	Systolic Blood Pressure
BUN	Blood Urea Nitrogen
PTT	Partial Thromboplastin Time
INR	International Normalized Ratio
LOS	Length of Stay
BIDMC	Beth Israel Deaconess Medical Center
VIF	Variance Inflation Factor
